# The influence of environmental temperatures and air humidity in the maintenance of the sterility of materials sterilized in different wraps

**DOI:** 10.1186/1753-6561-5-S6-P311

**Published:** 2011-06-29

**Authors:** CQDM Bruna, KU Graziano, FM Gomes Pinto

**Affiliations:** 1 Escola de Enfermagem, Universidade de São Paulo, São Paulo, Brazil

## Introduction / objectives

One of the variables which interfere in the maintenance of sterility in the materials is the storage. There are many official and not-official recommendations for temperature and air humidity control in the storage area, however without any theoretical or experimental basis. Some hospitals do not have a system that makes the control of temperature and humidity possible, and, in most cases, the sterilizing chambers are next to the storage area, constantly releasing heat and water vapour. Considering that one of the functions of the wraps is to maintain the sterility of the content, even under adverse conditions, the recommendations to have an environmental control in the storage area, a priori, would have a secondary importance. For these reason the doubt about the real importance of the temperature and humidity in the contamination of the materials stored after the autoclave sterilization arise.

## Methods

Therefore, an experiment was developed in which boxes with surgical instruments and cylinders carriers were packed in cotton sheets, crepe paper, SMS and surgical-grade paper, sterilized, and intentionally contaminated externally with *Serratia marcescens* 10^6^and stored in an environment with temperature around 35°C and air humidity around 75%. This group was compared with another group, the negative control, stored in temperature around 20 °C and air humidity around 60%. After a period of 30 days of storage, the carriers were removed from the boxes and incubated.

## Results

No bacterial growth was detected in any of the samples.

## Conclusion

The experiment results allowed concluding that the high temperature and high air humidity do not interfere in the barrier efficiency of the packs.

## Disclosure of interest

None declared.

